# Supplementary material for: Association between Perfluoroalkyl and Polyfluoroalkyl Substances and Women’s Infertility, NHANES 2013–2016
Source: Int J Environ Res Public Health. 2022 Nov 20;19(22):15348. doi: 10.3390/ijerph192215348 (PMC9692248; doi:10.3390/ijerph192215348)
Supplement: Supplementary file 1 [file ijerph-19-15348-s001.zip › Supplementary File S1.pdf]

Association between Perfluoroalkyl and Polyfluoroalkyl Substances and Women's Infertility, NHANES 2013–2016

Yuxuan Tan [1,†], Zurui Zeng [1,2,†], Huanzhu Liang [1], Xueqiong Weng [1,3], Huojie Yao [1], Yingyin Fu [1], Yexin Li [1], Jingmin Chen [1], Xiangcai Wei [1,2,*] and Chunxia Jing [1,4,*]

1 Department of Preventive Medicine and Public Health, School of Medicine, Jinan University, No. 601 Huangpu Ave West, Guangzhou 510632, China
2 Guangdong Women and Children Hospital, Guangzhou Medical University, Guangzhou 510632, China
3 Guangzhou Center for Disease Control and Prevention, Guangzhou 510440, China
4 Guangdong Key Laboratory of Environmental Exposure and Health, Jinan University, Guangzhou 510632, China
* Correspondence: dxcwei@163.com (X.W.); jcxphd@gmail.com (C.J.); Tel.: +86-20-8522-0258 (C.J.); Fax: +86-20-8522-1343 (C.J.)
† These authors contributed equally to this article.

Section A: BKMR Sensitivity Analysis

We conducted a BKMR model using the default setting of the "kmbayes" function in R. The function of the BKMR was as follows:

$$Y_i = h(PFDE, PFHxS, PFNA, n\_PFOA, n\_PFOS, Sm\_PFOS) + \beta X_i + \varepsilon_i$$

where Y_i represents the binary outcome for women infertility i (i = 1,…,n), h is the exposure–response function among the six PFAS exposures, and X_i and β represent covariates and their coefficients, respectively. We estimated the posterior inclusion probability (PIP) for each of PFAS exposures, which can be considered as a measure of variable importance, the higher the PIP (closer to 1), the greater the importance for outcome in BKMR model.

We varied these tuning parameters for fitting the algorithm to assess whether results changed:

(1) no variable selection (*varsel = False*). The prior certainty that all exposures can influence the outcome, which assumed that all exposures may impact outcomes.

(2) changing the "r" parameter prior into Gamma prior (*r.prior = "gamma"*). The prior distribution of "r" was assigned to inverse-uniform prior ("invunif") in default, which area must be between 0 and 1. As the recommended as the author Bobb et al.[1], the PIPs may be sensitive to the prior specifications, changing the prior distribution into the Gamma distribution may have some impact for the Metropolis-Hastings (M-H) steps.

(3) modifying the shape parameters for beta prior using a Beta(100,900)

hyperprior to change the skeptical variable selection(*a.*p_0*=100, b.*p_0*=900*), which quantifies strong prior information that only 1/10 of the included exposure variables actually influence the outcomes of interest.

These changing of prior information simulated BKMR fitting under an unknowing situation, and similarity of results would imply that our results are solid.

Section B: Supplementary Tables and Figures

Table S1. Characteristics of participants from NHANES 2013–2016.

N	Overall	Infertility No	Infertility Yes	*p*-Value
	788	682	106	
Age				<0.001 ***
Mean (SD)	35.48 (8.86)	35.02 (8.96)	38.44 (7.60)	
Race/Ethnicity (%)				0.029 *
Mexican American	124 (15.7)	116 (17.0)	8 (7.5)	
Other Hispanic	82 (10.4)	74 (10.9)	8 (7.5)	
Non-Hispanic White	270 (34.3)	222 (32.6)	48 (45.3)	
Non-Hispanic Black	182 (23.1)	156 (22.9)	26 (24.5)	
Other Race	130 (16.5)	114 (16.7)	16 (15.1)	
BMI				1.000
Underweight or normal	261 (33.3)	226 (33.3)	35 (33.7)	
Overweight or obese	522 (66.7)	453 (66.7)	69 (66.3)	
Education level (%)				0.027 *
Less than High school	136 (17.3)	127 (18.6)	9 (8.5)	
High school graduate or AA degree	147 (18.7)	128 (18.8)	19 (17.9)	
College or above	505 (64.1)	427 (62.6)	78 (73.6)	
Serum Cotinine (%)				0.563
Under 10 ng/dL	611 (77.5)	526 (77.1)	85 (80.2)	
Above 10 ng/dL	177 (22.5)	156 (22.9)	21 (19.8)	
Drink status (%)				0.513
No	256 (32.5)	225 (33.0)	31 (29.2)	
Yes	532 (67.5)	457 (67.0)	75 (70.8)	
Ratio of family income to poverty, n (%)				0.257
Lowest (≤1.37)	286 (38.5)	253 (39.5)	33 (32.4)	
Medium (1.37~3.25)	220 (29.6)	190 (29.7)	30 (29.4)	
Highest (≥3.25)	236 (31.8)	197 (30.8)	39 (38.2)	
Ever Pregnant (%)				0.130
No	183 (23.2)	165 (24.2)	18 (17.0)	
Yes	605 (76.8)	517 (75.8)	88 (83.0)	
Physical activity (%)				0.271
No	369 (46.8)	320 (46.9)	49 (46.2)	
Moderate	194 (24.6)	162 (23.8)	32 (30.2)	
Vigorous	225 (28.6)	200 (29.3)	25 (23.6)	
Marriage status (%)				0.003 **
Married	367 (46.6)	303 (44.4)	64 (60.4)	
Never married	216 (27.4)	200 (29.3)	16 (15.1)	
Other Status	205 (26.0)	179 (26.2)	26 (24.5)	
Age when first menstrual period				0.323
Mean (SD)	12.57 (1.75)	12.60 (1.74)	12.42 (1.81)	

Note: * $p < 0.05$; ** $p < 0.01$; *** $p < 0.001$.

Table S2. The non-linear relationship summary of Generalized additive model.

	EDF	Ref. DF	Chi.sq	*p*-Value
Individual PFAS				
PFDE	2.316	2.905	6.144	0.141
PFHxS	1.429	1.756	4.802	0.118
PFNA	2.620	3.305	7.09	0.081
n-PFOA	1.000	1.001	6.801	0.009 **
n-PFOS	4.013	4.975	9.547	0.087
Sm-PFOS	2.975	3.746	9.67	0.040 *
Total PFAS				
∑PFOS	3.673	4.600	9.992	0.060

Note: * $p < 0.05$; ** $p < 0.01$. Estimate degree of freedom, EDF; reference degree of freedom, Ref. DF.

Table S3. The characteristic of age-stratified participants, NHANES, 2013–2016.

		Age Group		
N	Overall	under 35	over 35	*p*-Value
	788	377	411	
Self-report infertility				<0.001 ***
No	682 (86.5)	345 (91.5)	337 (82.0)	
Yes	106 (13.5)	32 (8.5)	74 (18.0)	
Race/Ethnicity (%)				0.212
Mexican American	124 (15.7)	47 (12.5)	77 (18.7)	
Other Hispanic	82 (10.4)	41 (10.9)	41 (10.0)	
Non-Hispanic White	270 (34.3)	134 (35.5)	136 (33.1)	
Non-Hispanic Black	182 (23.1)	90 (23.9)	92 (22.4)	
Other Race	130 (16.5)	65 (17.2)	65 (15.8)	
Cotinine (%)				0.320
Under 10 ng/dL	611 (77.5)	286 (75.9)	325 (79.1)	
Above 10 ng/dL	177 (22.5)	91 (24.1)	86 (20.9)	
Ratio of family income to poverty, n (%)				0.005 **
Lowest	286 (38.5)	151 (41.8)	135 (35.4)	
Medium	220 (29.6)	116 (32.1)	104 (27.3)	
Highest	236 (31.8)	94 (26.0)	142 (37.3)	
Drink status (%)				0.010 *
No	256 (32.5)	105 (27.9)	151 (36.7)	
Yes	532 (67.5)	272 (72.1)	260 (63.3)	

Education level (%)				0.205
Less than High school	136 (17.3)	56 (14.9)	80 (19.5)	
High school graduate or AA degree	147 (18.7)	75 (19.9)	72 (17.5)	
College or above	505 (64.1)	246 (65.3)	259 (63.0)	
BMI (%)				<0.001 ***
Underweight or normal	261 (33.3)	149 (39.6)	112 (27.5)	
Overweight or obese	522 (66.7)	227 (60.4)	295 (72.5)	
Physical activity (%)				0.184
No	369 (46.8)	165 (43.8)	204 (49.6)	
Moderate	194 (24.6)	94 (24.9)	100 (24.3)	
Vigorous	225 (28.6)	118 (31.3)	107 (26.0)	
Marriage status (%)				<0.001 ***
Married	367 (46.6)	134 (35.5)	233 (56.7)	
Never married	216 (27.4)	157 (41.6)	59 (14.4)	
Other Status	205 (26.0)	86 (22.8)	119 (29.0)	
Age when first menstrual period				0.374
Median [IQR]	12.57 (1.75)	12.51 (1.73)	12.62 (1.77)	
Ever Pregnant (%)				
No	183 (23.2)	146 (38.7)	37 (9.0)	<0.001 ***
Yes	605 (76.8)	231 (61.3)	374 (91.0)	

Note: * $p < 0.05$; ** $p < 0.01$; *** $p < 0.001$.

Table S4. The concentration distribution of serum PFAS strafing in ages [a].

.	**Age under 35**		**Age over 35**		
	**Median [IQR]**	**GM (95%CI)**	**Median [IQR]**	**GM (95%CI)**	***p*-Value [b]**
Individual PFAS					
PFDE	0.10 [0.07, 0.20]	0.135 (0.125, 0.146)	0.20 [0.07, 0.30]	0.164 (0.151, 0.178)	< 0.001 ***
PFHxS	0.60 [0.40, 1.10]	0.664 (0.608, 0.725)	0.60 [0.40, 1.00]	0.592 (0.547, 0.640)	0.080
PFNA	0.40 [0.40, 0.70]	0.432 (0.402, 0.462)	0.50 [0.30, 0.80]	0.517 (0.482, 0.555)	< 0.001 ***
n-PFOA	1.10 [0.70, 1.50]	0.982 (0.909, 1.061)	1.10 [0.70, 1.80]	1.086 (1.006,1.172)	0.245
n-PFOS	2.10 [1.30, 3.10]	2.040 (1.875, 2.219)	2.30 [1.30,3.80]	2.336 (2.138, 2.552)	0.023 *
Sm-PFOS	0.70 [0.40, 1.10]	0.638 (0.592, 0.688)	0.70 [0.40, 1.20]	0.720 (0.665, 0.780)	0.033 *
Total PFAS					

∑PFOS	2.80 [1.80, 4.30]	2.739 (2.530, 2.965)	3.20 [1.90, 5.00]	3.139 (2.887, 3.414)	0.018 *

Note: * $p < 0.05$; *** $p < 0.001$. [a]. All values of tertile was presented in medians. [b]. The differences in serum PFAS concentrations between the two groups were measured using the rank sum test.

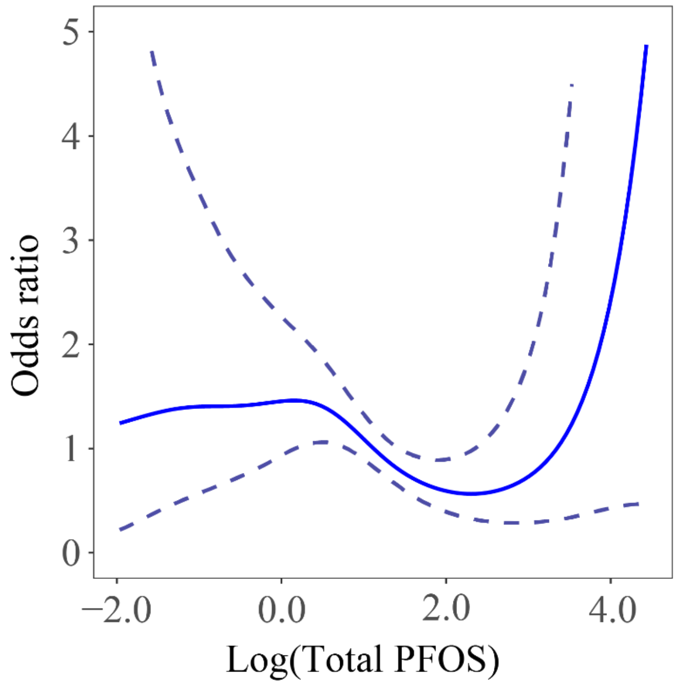

Figure S1. The effect (95% CI) of the total PFOS on infertility by GAM non-linear regression.

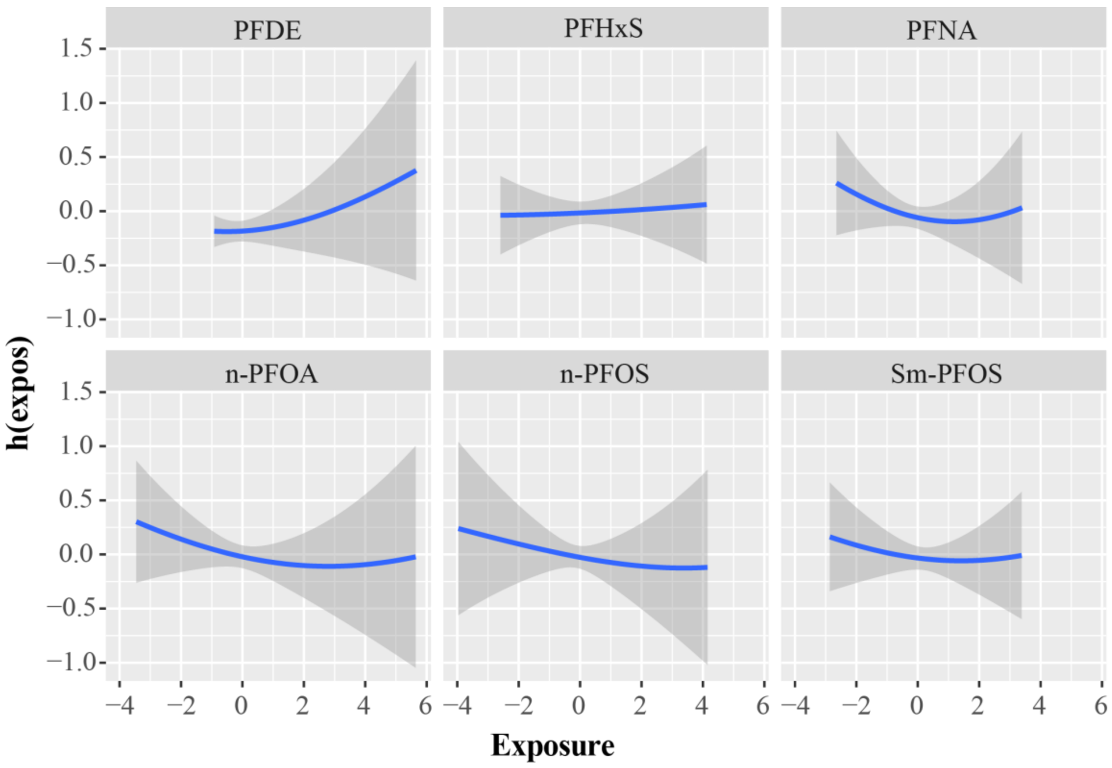

Figure S2. Univariate exposure-response functions and 95% confidence interval for each exposure fixed at the median using BKMR.

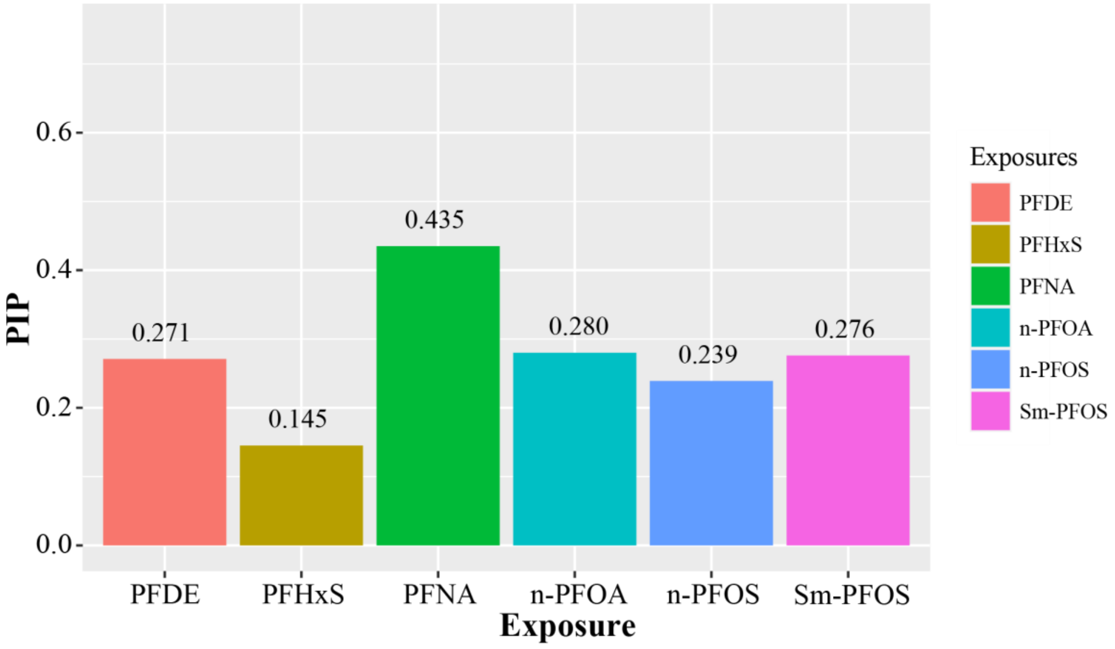

Figure S3. Posterior inclusion probabilities (PIPs) for each exposure, using Bayesian kernel machine regression (BKMR) model (N=788), NHANES, USA, 2014–2016.

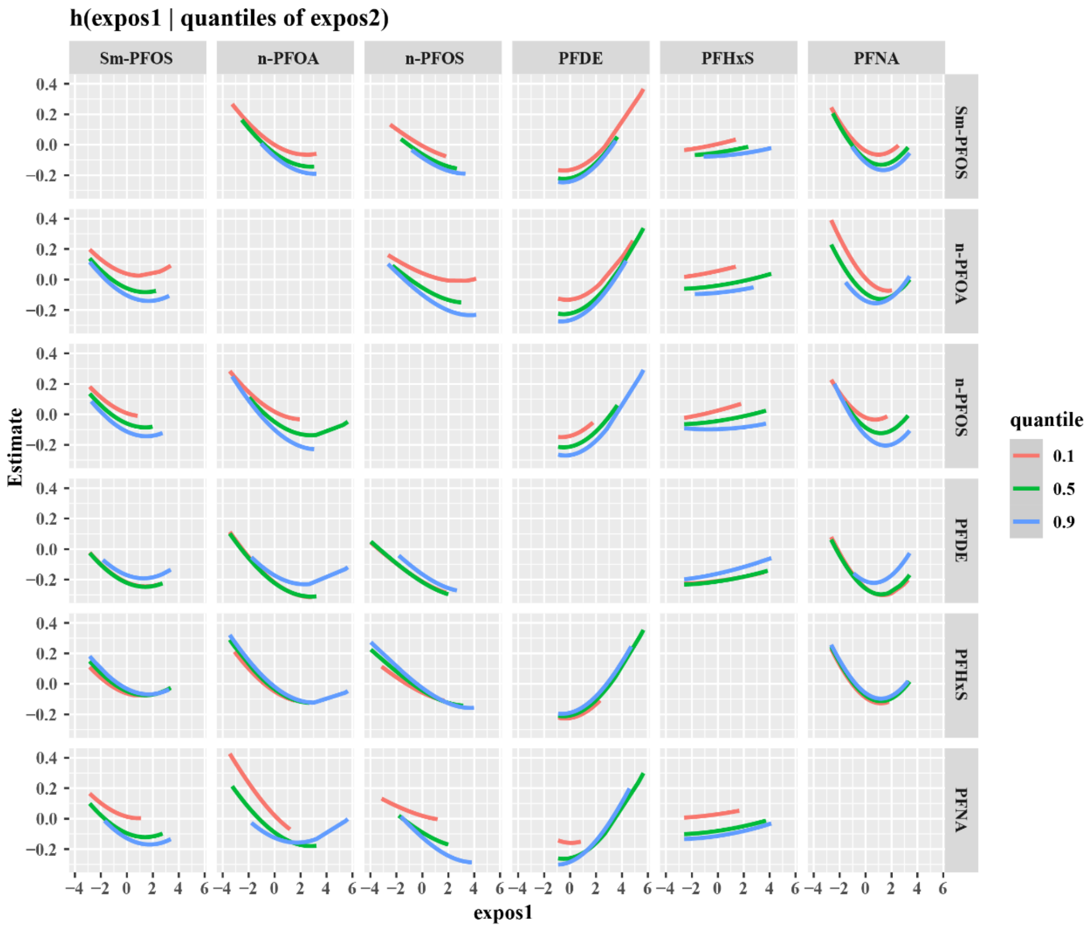

Figure S4. The trend of exposure-response in three quantiles, the function of a single exposure where the other exposure was fixed at 0.1, 0.5, 0.9 quantiles.

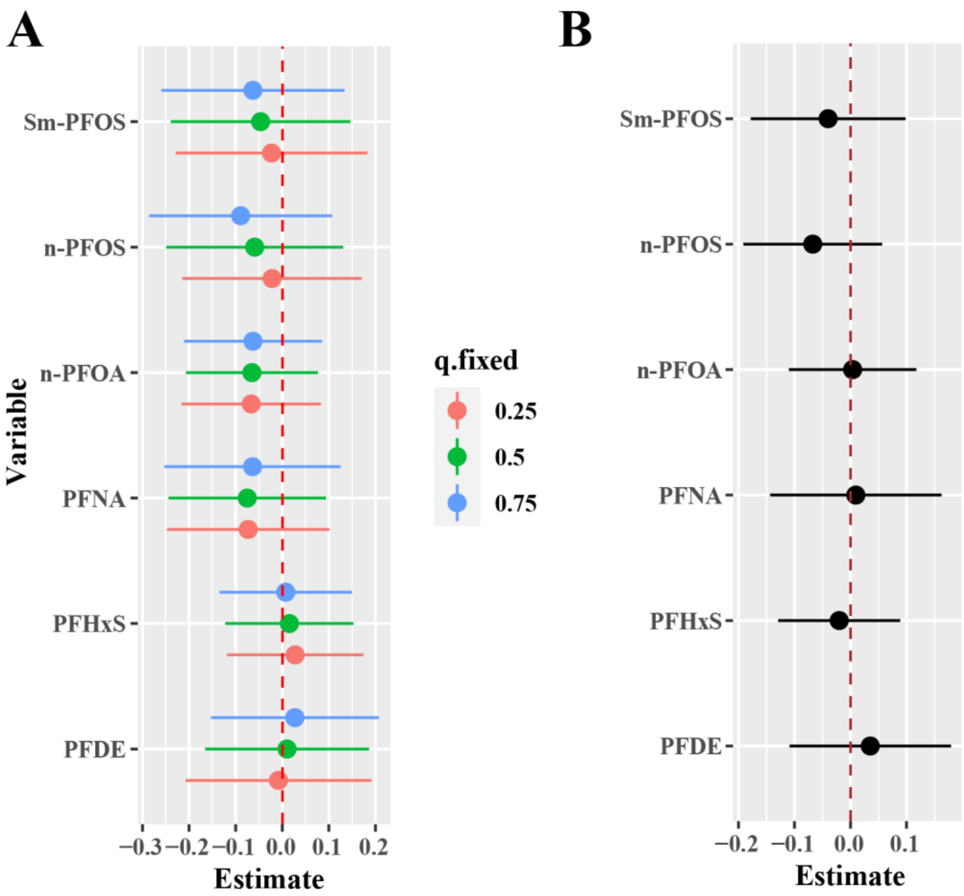

Figure S5. The single-exposure risk comparing using the three quantiles, whether the change in the 75th percentile is statistically significant compared with the 25th percentile, which can support the existence of potential interaction. Neither the (**A**) and (**B**) showed a potential interaction in PFAS.

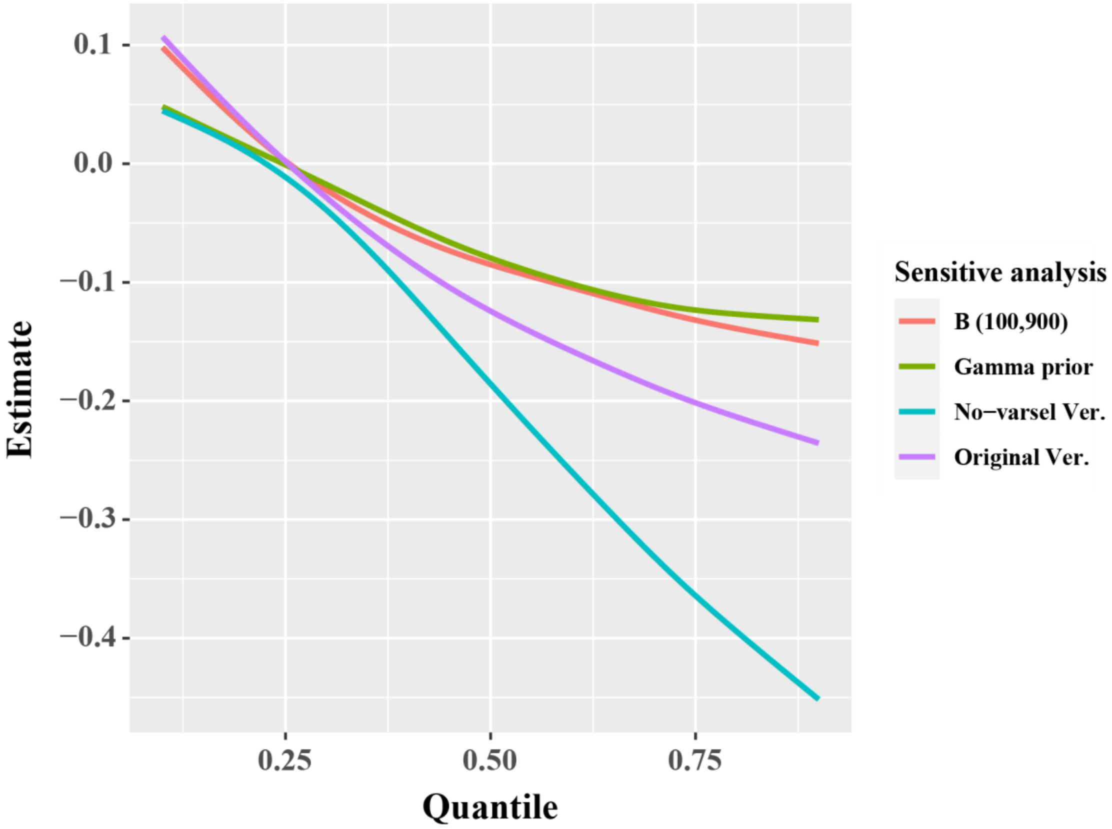

Figure S6. Comparison of the models from sensitivity analyses for BKMR. Violet: Main analysis, Sky-blue: No variable selection, Green: Gamma prior in the default setting, and Red: Very skeptical variable selection, using Beta (100,900) prior. Using GAM smooth to make the curve less glitchy.

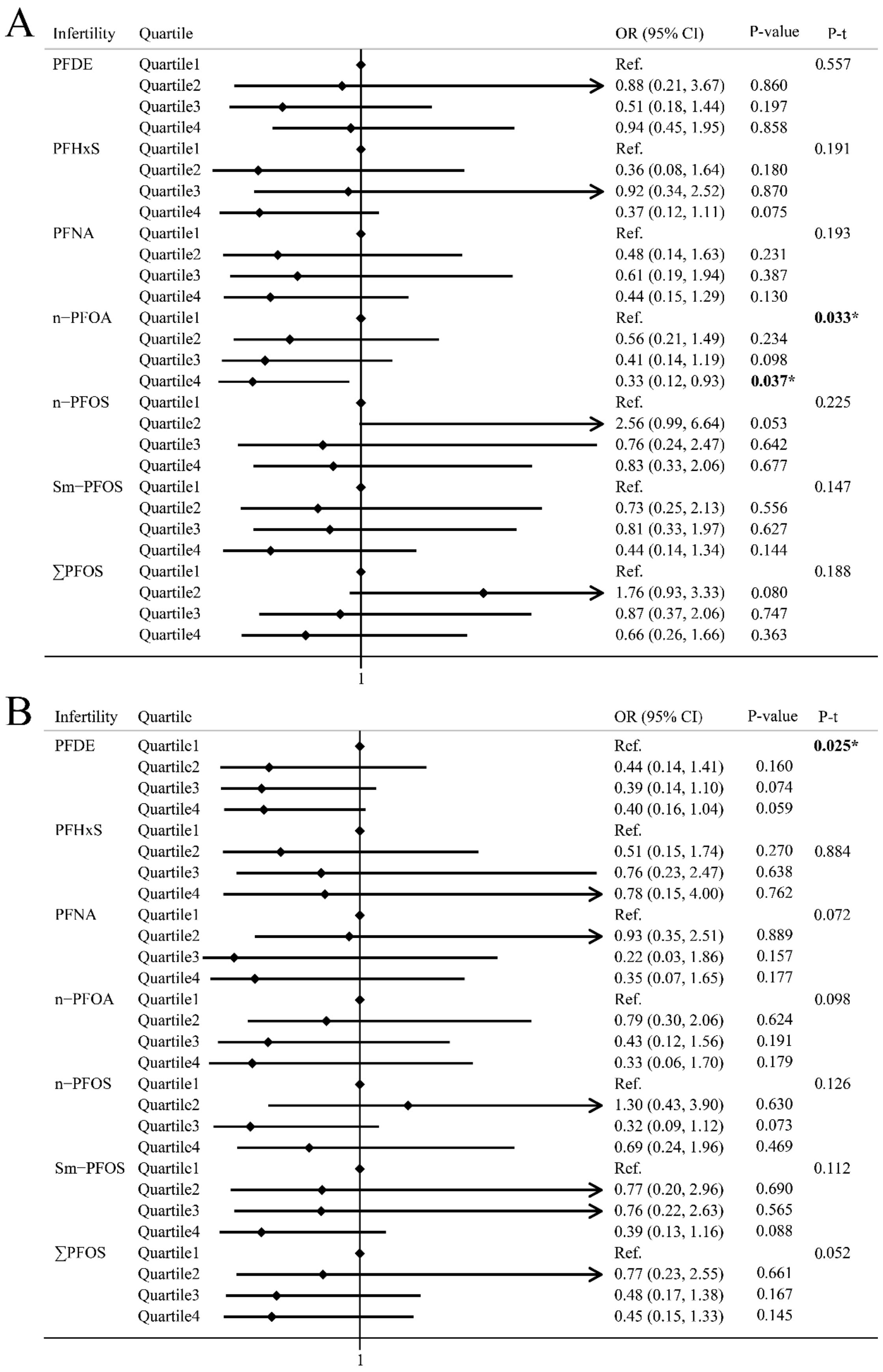

Figure S7. The subgroup analysis of age stratification. (**A**) showed women

younger between 35 to 50, and (**B**) shows women under 35 years old. Note: * $p <$ 0.05.

Reference

1. Bobb, J. F.; Valeri, L.; Claus Henn, B.; Christiani, D. C.; Wright, R. O.; Mazumdar, M.; Godleski, J. J.; Coull, B. A., Bayesian kernel machine regression for estimating the health effects of multi-pollutant mixtures. *Biostatistics* **2015**, 16, 493-508.
